# Presenting Visual Acuity and Ocular Comorbidity in Patients with Primary Open Angle Glaucoma in a Private Tertiary Eye Center in Nigeria

**DOI:** 10.5005/jp-journals-10008-1129

**Published:** 2013-01-15

**Authors:** Roseline Duke, Ayodele Akinye, Soter Ameh

**Affiliations:** Chief Consultant, Department of Ophthalmology, University of Calabar Teaching Hospital, Calabar, Cross River State, Nigeria; Senior Registrar, Eye Foundation Group of Hospitals, Ikeja, Lagos, Nigeria; Consultant and Community Physician, Department of Community Medicine, University of Calabar Teaching Hospital, Calabar, Cross, River State, Nigeria; School of Public Health, University of the Witwatersrand, Johannesburg South Africa

**Keywords:** Ocular comorbidity, Visual acuity, Primary open angle glaucoma.

## Abstract

**Purpose:** To determine the presenting visual acuity (VA) of patients with primary open angle glaucoma (POAG) and the relationship to ocular comorbidity.

**Materials and methods:** A retrospective case note audit was undertaken in Eye Foundation Hospital, Calabar, between 1st January 2010 and 31st June 2011. The case notes of all newly presenting patients diagnosed with POAG were retrieved and data were extracted for analysis. Inclusion criteria for glaucoma was defined.

**Results:** Out of a total of 320 new patients, 88 patients were diagnosed with POAG, with a prevalence of 27.5% (95% CI: 22.7-32.7). The mean age for males is 57.1 ± 8.8 while that of females is 52.6 ± 11.2 and the total mean of 55.8 ± 9.7. Of the 88 patients, there were 84 self referrals [95.5% (95% CI: 88.8-98.7)], of these, 45, 53.6% (95% CI: 42.4-64.5) were for second opinion. There was no statistical significance between the gender, education and occupation and the level of VA seen on presentation.

The best corrected visual acuity (BCVA ) in the right and left eye respectively, was significantly (p < 0.0001) related to the cup disk ratio. Ocular comorbid conditions were seen in 19 (22%) patients in the study. The presenting BCVA was significantly related to the presence of ocular comorbidity in the best eye. Comorbidity was not strongly related to the age of the patients.

**Conclusion:** The course of glaucomatous progression is highly variable, identifying factors that not only predict progression but influence the VA and ocular health of the eye can help to guide clinical practice and patient treatment and monitoring.

**How to cite this article:** Duke R, Akinye A, Ameh S. Presenting Visual Acuity and Ocular Comorbidity in Patients with Primary Open Angle Glaucoma in a Private Tertiary Eye Center in Nigeria. J Current Glau Prac 2013;7(1):6-10.

## INTRODUCTION

In Nigeria, it is estimated that 1,130,000 individuals aged greater than 40 years are blind and 4.25 million adults in this same group have moderate to severe visual impairment or blindness.^1-3^ Further, 84% of this visual loss is avoidable. The two main causes of blindness and visual impairment in Nigerian adults are: Cataract with prevalence of 1.8% (95% CI: 1.57-2.05) and glaucoma with a prevalence of 0.7% (95% CI: 0.6-0.9).^3-5^

Glaucoma is a progressive optic nerve disease that results in characteristic damage to the optic nerve and loss of retinal ganglion cells leading to progressive loss of function or visual field damage.^6,7^ Primary open angle glaucoma (POAG) is a complex disease and accounts for 70% of all cases of glaucoma.^7^ About 57% of cases of POAG, remains are undiagnosed in Nigeria and a similar figure in Tanzania and South Africa.^8,9^ Glaucoma progresses from undetectable to asymptomatic to functional impairment to blindness.^10^

Understanding the risk factors that predispose to the development of POAG and blindness from the disease is essential for early identification of the disease as well as early referral of such cases. Appropriate treatment and follow-up are all necessary for the prevention of irreversible blindness from POAG and importantly, the preservation of a good quality of life for the patient. This retrospective hospital-based study was undertaken in a private tertiary eye facility to determine the presenting visual acuity (VA) of patients with POAG and the relationship with ocular comorbid conditions.

## MATERIALS AND METHODS

A retrospective case note audit was undertaken in Eye Foundation Hospital, located in the city of Calabar Cross River State, between 1st January 2010 and 31st June 2011. Eye Foundation hospital Calabar is a tertiary eye center, offering subspecialty eye services including glaucoma and vitreoretinal surgical services. The case notes of all the patients that were diagnosed with POAG were retrieved and studied during the study period. Data was extracted from the case notes, filled into a questionnaire and transferred to an excel sheet.

Inclusion criteria were adults (greater than 15 years of age) who presented for the first time to the facility with diagnosis of POAG made in our hospital based on characteristic glaucomatous changes seen on stereoscopic assessment of the optic nerve head using a 78D lens, in combination with a central visual field measurement using a Kowa 2008 model, with a mean deviation of at least -5dB in the presence of an open angle on gonioscopy and the absence of any other secondary causes of a raised intraocular pressure. Further all participating patients had a dilated fundus examination using a binocular indirect ophthal-moscopy and a three mirror lens examination.

Sociodemographic variables included age, sex, occupation and level of education. The best corrected VA (BCVA) in the right and left eye respectively on the first visit as well as the cup disk ratio of the patients was extracted. The level of statistical significance was set at 95% (p < 0.05) and statistical analysis was done using STATA version 10 statistical package. Chi-square test (Fisher's exact test where expected cell count was less than 5) was used to test the relationship or association between categorical variables.

Ethical approval for the study was obtained from the Institutional Ethics Committee of Eye Foundation Hospital.

## RESULTS

Out of a total of 320 new hospital presentations seen within the period of study, 88 were presenting patients who we diagnosed with POAG in our hospital, resulting in a POAG prevalence of 27.5% (95% CI: 22.7-32.7). Of the 88 patients diagnosed with POAG, there were 84 self referrals [95.5% (95% CI: 88.8-98.7)]. Among the self-referred patients, 45 were for second opinion after being previously diagnosed with POAG at another health facility [53.6% (95% CI: 42.4-64.5)]. Among the self referred, previously diagnosed POAG patients who consulted at the Eye Foundation for second opinion, 28 (62%) presented with a VA worse than 6/18 while 17 (38%) had best corrected vision between 6/6 to 6/18. Of the four (5%) referred from other eye health facilities, three (75%) had best corrected VAs worse than 6/18 (Table 1).

The mean age for males is 57.1 ± 8.8 while that of females is 52.6 ± 11.2 and the total mean of 55.8 ± 9.7 with the independent t-test of 1.962 and p-value of 0.053. Males [64 (72%)] were significantly more than females [24 (27%)] in this study population. The age group of 40 to 45 years presented with 15 (17%) patients which was the least, while the age group with the highest presentation to the facility was in the 46 to 55 age category 30 (34%); however, the highest number of patients with severe visual impairment and blindness was seen between the 56 to >66 years age group (Table 2). There was no significant relationship between gender and VA in the better eye.

Seventy-four percent (20/27) of patients with a presenting BCVA worse than or equal to 6/60 in one eye were tertiary educated. There was no statistically significant relationship between respondents' education and VA in the better eye. Skilled workers made up 78% (n = 69) of the study population, and there was no statistical significance between the occupation and the level of BCVA at presentation.

Topical medications were used by 61 (69.3%) patients on presentation of these, 42 (69%) were on combination therapy of Travaprost 0.004: Timolol 0.25 (duotrav), 12 (20%) were on triple therapy of duoatrav and azopt (Brinzolamide 1% topical carbonic anhydrase inhibitor) and 7 (11%) had received tablets of systemic carbonic anhyrdase inhibitor acetazolamide (diamox) utilized for a period of at least 4 weeks as at presentation. Twenty-seven (30.7) patients were not on any topical medications as at presentation.

**Table 1:** **Table 1:** Characteristics of POAG patients

*Variables*		*Number*		*Proportion (%)*		*95% CI*	
*POAG*							
Yes		88		27.5		22.7-32.7	
No		232		72.5			
Total		320		100			
*Referral for POAG*							
Self		84		95.5		88.8-98.7	
Health facilities		4		4.5			
Total		88		100			
*Self-referred patient*							
Second opinion		45		53.6		42.4-64.5	
Remainder		39		46.4			
Total		84		100			
*Self-referred patients for second opinion*							
VA worse than 6/18		28		62.2		46.5-76.2	
VA 6/6-6/18		17		37.8			
Total		45		100			
*Health facility referred patients*							
Best corrected VA worse than 6/18		3		75.0		19.4-99.4	
Best corrected VA 6/6-6/18		1		25.0			
Total		4		100			

**Table 2:** **Table 2:** Relationship between age group and VA in the better eye

*Age (years)*		*VA better eye n (%)*										Total		*Fisher's test p-value*	
		6/12		6/12-6/18		6/18-6/60		6/60-3/60		<3/60					
40-45		7 (46.7)		2 (13.3)		3 (20.0)		3 (20.0)		0 (0.0)		15 (17.0)			
46-55		12 (40.0)		7 (23.4)		6 (20.0)		4 (13.3)		1 (3.3)		30 (34.0)		0.117	
56-65		5 (23.8)		1 (4.8)		6 (28.5)		8 (38.1)		1 (4.8)		21 (24.0)			
≥66		3 (13.6)		6 (27.3)		3 (13.6)		6 (27.3)		4 (18.2)		22 (25.0)			
Total		27 (30.7)		16 (18.2)		18 (20.4)		21 (23.9)		6 (6.8)		88 (100)			

It was seen that, nine (10%) patients had previously undergone trabeculectomy, with 1 (1%) patient presenting with a history of previous argon laser trabeculoplasty, no patient had undergone G probe laser procedure and 78 (89%) had no other surgical intervention. Regarding systemic diseases, 8 (9%) had well controlled systemic hypertension and were on oral antihypertensive medications while 12 (14%) patients reported to be having systemic diabetes mellitus. Only one patient had a glycocylated hemoglobin result within 4.3 to 6.7%. No patient in our series had a history of smoking.

A positive family history of POAG was seen in 34 (39%) patients. There was no statistical significance seen between BCVA, the age group and the central visual fields in both eyes.

Ocular comorbid conditions were seen in 19 (22%) patients in the study. The presenting BCVA was significantly related to the presence of ocular comorbidity in the best eye. However, comorbidity was not strongly related to the age of the patients (Tables 3 and 4).

The BCVA in right eyes was significantly (p < 0.0001) related to the cup disk ratio (Table 5). A cup disk ratio exceeding 0.8 was more likely to be associated with a visual BCVA of 6/60 or worse compared to a cup disk ratio less than or equal to 0.7. The finding was the same for the left eye (Table 6).

**Table 3:** **Table 3:** Comorbidity by BCVA in the best eye

*Presenting VA in the best eye*		*Ocular comorbidity*								*Total no. (%)*		*Fisher's test p-value*	
		*Diabetic eye disease*		*Significant lens opacity*		*CRVO*		*AMD*					
6/12		2 (25.0)		0 (0.0)		0 (0.0)		1 (50.0)		3 (15.7)		0.003	
6/12-6/18		5 (62.5)		0 (0.0)		0 (0.0)		0 (0.0)		5 (26)			
6/18-6/60		0 (0.0)		0 (0.0)		0 (0.0)		0 (0.0)		0 (0)			
6/60-3/60		1 (12.5)		3 (37)		1 (100.0)		0 (0.0)		4 (21)			
<3/60		0 (0.0)		5 (63)		0 (0.0)		1 (50.0)		6 (31.5)			
Total		8 (42.1)		8 (42.1)		1 (5.3)		2 (10.5)		19 (100)			

**Table 4:** **Table 4:** Comorbidity in the age groups

*Presenting VA in the best eye*		*Ocular comorbidity*								*Total no. (%)*		*Fisher's test p-value*	
		*Diabetic eye disease*		*Significant lens opacity*		*CRVO*		*AMD*					
36-45		2 (25.0)		1 (12.5)		0 (0.0)		0 (0.0)		3 (15.8)		0.957	
46-55		3 (37.5)		2 (25.0)		0 (0.0)		1 (50.0)		6 (31.6)			
56-65		2 (25.0)		3 (37.5)		0 (0.0)		0 (0.0)		5 (26.3)			
≥66		1 (12.5)		2 (25.0)		1 (100)		1 (50.0)		5 (26.3)			
Total		8 (42.1)		8 (42.1)		1 (5.3)		2 (10.5)		19 (100)			

**Table 5:** **Table 5:** Relationship between VA in right eye and CD ratio in right eye

*VA right eye*		*CD ratio right eye (%)*								*Total no. (%)*		*Fisher's test p-value*	
		*0.0-0.5*		*0.6-0.7*		*0.8-0.9*		*1.0*					
6/12		3 (21.4)		8 (53.3)		8 (18.6)		0 (0.0)		19 (21.6)			
6/12-6/18		3 (21.4)		4 (26.7)		6 (14.0)		0 (0.0)		13 (14.8)		0.001	
6/18-6/60		4 (28.6)		2 (13.3)		10 (23.3)		2 (12.5)		18 (20.4)		Significant	
6/60-3/60		3 (21.4)		0 (0.0)		14 (32.5)		9 (56.3)		26 (29.6)			
<3/60		1 (7.2)		1 (6.7)		5 (11.6)		5 (31.2)		12 (13.6)			
Total		14 (15.9)		15 (17.0)		43 (48.9)		16 (18.2)		88 (100.0)			

**Table 6:** **Table 6:** The relationship between left VA and CDR in the left eye

*VA left eye*		*CD ratio left eye (%)*								*Total no. (%)*		*Fisher's test p-value*	
		*0.0-0.5*		*0.6-0.7*		*0.8-0.9*		*1.0*					
6/12		2 (20.0)		5 (29.4)		11 (25.0)		0 (0.0)		19 (21.6)			
6/12-6/18		2 (20.0)		4 (23.5)		6 (13.6)		0 (0.0)		13 (14.8)		0.001	
6/18-6/60		3 (30.0)		6 (35.3)		7 (15.9)		1 (5.9)		18 (20.4)		Significant	
6/60-3/60		1 (10.0)		1 (5.9)		7 (15.9)		3 (17.6)		26 (29.6)			
<3/60		2 (20.0)		1 (5.9)		13 (29.6)		13 (76.5)		12 (13.6)			
Total		10 (11.4)		17 (19.3)		44 (50.0)		17 (19.3)		88 (100.0)			

## DISCUSSION

The management of primary open angle glaucoma is challenging. The natural history of the lack of symptoms in POAG plays a large role in delaying its detection and diagnosis. Typically, POAG is slowly progressive, remaining asymptomatic until late. By the time POAG becomes symptomatic, severe and irreversible damage has usually occurred to the visual field in one or both eyes. The rate of progression of the visual field defect varies in patients, and treatment of the glaucoma may not completely halt the visual field loss.^11^ Some patients progress despite aggressive therapy.^12^

Self referrals and especially second opinion seekers presented with visual impairment. It has been seen that majority of patients that have POAG on their first hospital visit present with visual impairment.^13^ Self referrals may result from health education about glaucoma from family members, as 39% of patients had a family history of the disease or glaucoma case detection and referral from primary or secondary health facilities. Further, self-referred second opinion seekers, may be presenting because POAG initially affects the peripheral vision, making it difficult for an individual to comprehend the gravity to the threat to the central vision, hence the disbelief as to the capability of the disease to cause blindness. Further, reports have shown that, compliance with poor follow-up visits for glaucoma has been associated with markers for early disease.^14^ More so, by the time painless irreversible visual impairment occurs, patients may feel they are in a dilemma and seek second opinions to refute or confirm what has been told to them previously about the disease. It is also reasonable to think that the occurrence or progression of an ocular morbid condition could worsen the vision, thereby making the patient seek help afresh.

Primary open angle glaucoma has an insidious onset which begins in early adulthood and results in blindness at a much later time.^15^ The number of years with primary open angle glaucoma in which the patient has to manage the disease has being said to be approximately 30 to 45 years.^10^ Hospital eye care systems will need to put in place systems to prevent hospital fatigue in patients with such a chronic disease as POAG. However, suggestions to improve compliance, might focus on improved communication of the seriousness of the disease and improvement in clinic waiting time.^14^ These may reduce the desire for unnecessary second opinions. In addition, strategies using the identification of risk factors for the development of glaucoma and glaucoma blindness can be used to provide guidelines for targeting at-risk groups to improve early glaucoma detection, treatment and follow-up. These are currently, the most powerful tools for preventing blindness and low vision in this predominantly asymptomatic disease in its early stages.^16^ In Nigeria, some of these strategies are utilized within tertiary eye hospital settings. Glaucoma prevention and control programs have not been instituted in the country.

In the 2010 projected world population, 51.5% of those over 40 years are female. Currently, 59% of those who have glaucoma are females.^7^ More males were seen with glaucoma. In our study, the age group with the highest hospital presentation can be seen in Table 2 that suggests an age group which is economically productive and active, an age group where hospital attendance may be a challenge due to time constraints.

This study is limited in that, it is hospital based and not a population or multicenter survey; this probably explains why we have more males than female. In our environment, men are economically more empowered than women and maybe more likely to afford private service. It is expected that patients with higher educational status would earn more and therefore better able to utilize private eye care services.

The risk of the progression of POAG increases with age, while the prevalence of POAG tends to double with each decade of life after 40 years.^8,17,18^ Ocular comorbid conditions may be partly responsible for the compromise to the vision that is seen in POAG patients. These conditions may compromise vision in POAG patients regardless of the age. Diabetic eye disease especially clinically significant diabetic macular odema and proliferative diabetic retinopathy as well as significant lens opacity in our study was seen equally to be the two most common causes followed by age-related macular degeneration. Visual loss from these causes are treatable. Ocular comorbid conditions should be sought for in all patients with a visual reduction in primary open angle glaucoma regardless of the age of the patient.

## CONCLUSION

An eye examination including a thorough anterior segment and fundus examination is critical in explaining the cause of visual impairment in patients with POAG. It is necessary to detect associated ocular comorbid conditions in the eye and treat, so as to reduce its contribution to the visual debility of POAG.

## References

[1] Rabiu MM, Kyari F, Ezelum C, Elhassan E, Sanda S, Murthy GV, Sivasubramaniam S, Glibert C, Abdull MM, Abiose A (2012). Review of the publications of the Nigeria national blindness survey: methodology, prevalence, causes of blindness and visual impairment and outcome of cataract surgery.. Ann Afr Med.

[2] Quigley HA (1996). Number of people with glaucoma worldwide.. Br J Ophthalmol.

[3] Thylefors B, Negrel AD (1994). The global impact of glaucoma.. Bull World Health Organ.

[4] Quigley HA, Broman AT (2006). The number of people with glaucoma worldwide in 2010 and 2020. Br J Ophthalmol.

[5] Ashaye AO 12th Faculty of Ophthalmology Lecture, National Postgraduate Medical College of Nigeria 2010 Glaucoma blindness: facts, fancies and fables. [place unknown]: [publisher unknown]; [date unknown]..

[6] Varma R, Tielsch JM, Quigley HA, Hilton SC, Katz J, Spaeth GL, Sommer A (1994). Race-, age-, gender- and refractive error-related differences in the normal optic disc.. Arch Ophthalmol.

[7] Sathyamangalam RV, Paul PG, George R, Baskaran M, Hemamalini A, Madan RV, Augustian J, Prema R, Lingam V (2009). Determinants of glaucoma awareness and knowledge in urban Chennai.. Indian J Ophthalmol.

[8] Kosoko-Lasaki O, Gong G, Haynatzki G, Wilson MR (2006). Race, ethnicity and prevalence of primary open-angle glaucoma.. J Natl Med Assoc.

[9] Rotchford AP, Kirwan JF, Muller MA, Johnson GJ, Roux P (2003). Temba glaucoma study: a population-based cross-sectional survey in urban South Africa.. Ophthalmology.

[10] Weinreb RN, Khaw PT (2004). Primary open-angle glaucoma.. Lancet.

[11] Leske MC, Heijl A, Hyman L, Bengtsson B, Komaroff E (2004). Factors for progression and glaucoma treatment: the Early Manifest Glaucoma Trial.. Curr Opin Ophthalmol.

[12] Oliver JE, Hattenhauer MG, Herman D, Hodge DO, Kennedy R, Fang-Yen M, Johnson DH (2002). Blindness and glaucoma: a comparision of patients progressing to blindness from glaucoma with patients maintaining vision.. Am J Ophthalmol.

[13] Awoyesuku EA, Ejimadu CS (2012). Visual disability in newly diagnosed primary open angle glaucoma (POAG) patients in a tertiary hospital in Nigeria.. Niger J Med.

[14] Kosoko O, Quigley HA, Vitale S, Enger C, Kerrigan L, Tielsch JM (1998). Risk factors for noncompliance with glaucoma follow-up visits in a residents’ eye clinic.. Ophthalmology.

[15] Luo RJ, Liu SR, Tian Z, Zhu WH, Zhuo YH, Liao RD (2011). Rehabilitation of vision disorder and improved quality of life in patients with primary open angle glaucoma.. Chin Med J (Engl).

[16] Pan Y, Varma R (2011). Natural history of glaucoma.. Indian J Ophthalmol.

[17] Tielsch JM, Somner A, Katz J, Royall RM, Quigley HA, Javitt J (1991). Racial variations in the prevalence of primary open angle glaucoma. The Baltimore Eye Surgery.. JAMA.

[18] Ashaye AO (2003). Clinical features of primary glaucoma in Ibadan.. Nig J Ophthalmol.

